# Quantitative Standards of 4‐*O*‐Acetyl‐ and 9‐*O*‐Acetyl‐*N*‐Acetylneuraminic Acid for the Analysis of Plasma and Serum

**DOI:** 10.1002/cbic.202100662

**Published:** 2022-01-18

**Authors:** Jack Cheeseman, Concepcion Badia, Rebecca I. Thomson, Gunter Kuhnle, Richard A. Gardner, Daniel I. R. Spencer, Helen M. I. Osborn

**Affiliations:** ^1^ School of Pharmacy University of Reading Whiteknights Reading RG6 6AD UK; ^2^ Department of Food and Nutritional Sciences University of Reading, Whiteknights Reading RG6 6AH UK; ^3^ Ludger Ltd. Culham Science Centre Abingdon OX14 3EB UK

**Keywords:** acetylated sialic acid, carbohydrates, DMB assay, qNMR spectroscopy, UHPLC

## Abstract

*N*‐Acetylneuraminic acid (sialic acid, Neu5Ac) is one of a large, diverse family of nine‐carbon monosaccharides that play roles in many biological functions such as immune response. Neu5Ac has previously been identified as a potential biomarker for the presence and pathogenesis of cardiovascular disease (CVD), diabetes and cancer. More recent research has highlighted acetylated sialic acid derivatives, specifically Neu5,9Ac_2_, as biomarkers for oral and breast cancers, but advances in analysis have been hampered due to a lack of commercially available quantitative standards. We report here the synthesis of 9‐*O*‐ and 4‐*O*‐acetylated sialic acids (Neu5,9Ac_2_ and Neu4,5Ac_2_) with optimisation of previously reported synthetic routes. Neu5,9Ac_2_ was synthesised in 1 step in 68 % yield. Neu4,5Ac_2_ was synthesised in 4 steps in 39 % overall yield. Synthesis was followed by analysis of these standards via quantitative NMR (qNMR) spectroscopy. Their utilisation for the identification and quantification of specific acetylated sialic acid derivatives in biological samples is also demonstrated.

## Graphical abstract

**Synthesised acetylated** derivatives of *N*‐acetylneuraminic acid, Neu5,9Ac_2_ and Neu4,5Ac_2_, alongside commercially available Neu5Ac and Neu5Gc were utilised as standards for the quantitative analysis of these derivatives in plasma and serum samples. Multiple derivatives could be detected in one assay, which exhibited high specificity and low limits of detection and quantitation.

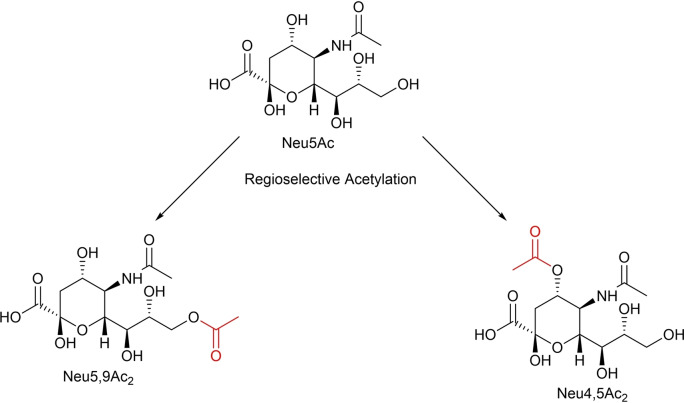

## Introduction

**Figure 1:**
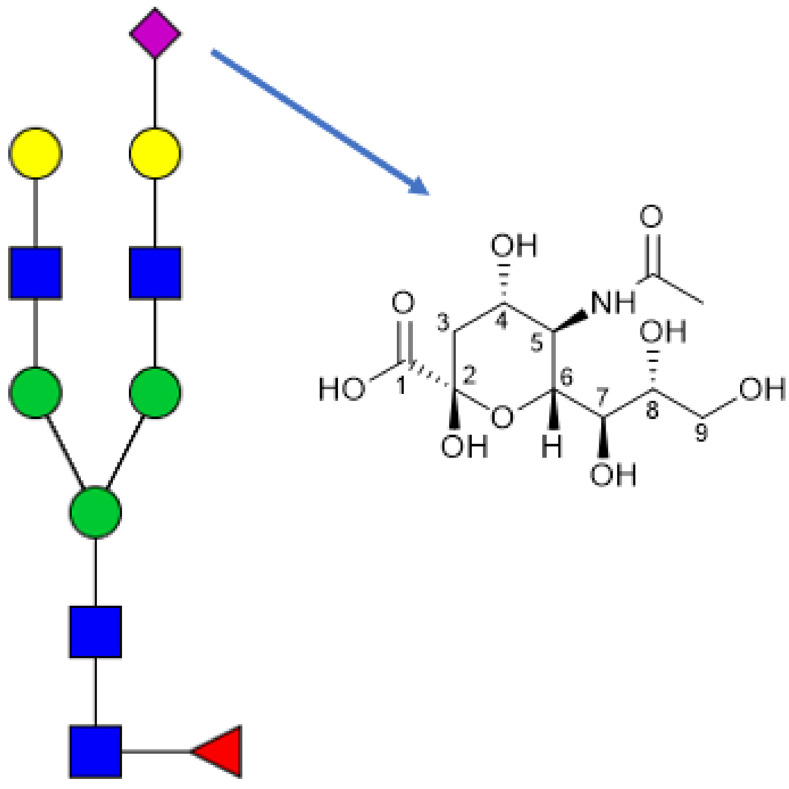
*N*‐Glycan with *N‐*Acetylneuraminic acid as the terminating unit of one branch.

Neu5Ac is a nine‐carbon backbone monosaccharide with a carboxylic acid functional group (Figure 1) and is one of a family of over fifty neuraminic acids (sialic acids).^[1]^ Structural diversity arises from functionalisation at the 5‐position (acetyl, glycolyl, lactyl, alcohol groups) as well as at one or more of the five hydroxyl positions (acetyl, methyl, phosphate, sulfate).^[2]^ Further to this, glycosidic bonds between the 2‐position of sialic acids and different carbohydrate frameworks allow for the creation of a range of different sialic acid‐containing glycans. Lastly, different linkages of sialic acid (α‐2,3, α‐2,6, α‐2,8, α‐2,9) increase this diversity further still.^[3]^

Sialic acids are generally located on *N*‐glycans as the terminating unit (Figure 1),^[4]^ forming parts of glycoconjugates such as glycoproteins and glycolipids. Sialic acids can also be found as internal residues of these glycans, particularly when an α‐2,8 linkage is present, and as part of polysialic acid chains.^[5]^ In far smaller quantities, free or unbound Neu5Ac is present in biological fluids. In primates and other mammals, the two major species found are Neu5Ac and Neu5Gc (Neu5Gc is not naturally produced in humans). Small quantities of other derivatives of Neu5Ac have been identified, for example Neu5,9Ac_2_ ^[6]^ in humans and a wider variety of sialic acids, including Neu4,5Ac_2_, in other species.[^6^, ^7^]

Neu5Ac has many important biological functions in humans including modulation of the immune system^[8]^ and, due to the overall negative charge of the molecule, prevention of erythrocyte aggregation and thereby clotting.^[9]^ Cancer cells exhibit overexpression of cell surface Neu5Ac to avoid immune system detection and act as a biological mask, this behaviour aids cancer cell proliferation and promotes angiogenesis.^[10]^ Neu5Ac is not the only neuraminic acid of biological importance. Neu5,9Ac_2_ has also been indicated to play roles in modulating the immune system and stability of glycoproteins. Possible roles in cancer development, autoimmune conditions, and infection have also been articulated.^[11]^ These roles are owed to different characteristics such as increased hydrophobicity, size and hydrogen bonding compared to Neu5Ac due to the presence of the additional acetyl functional group.^[12]^ Expression of Neu5,9Ac_2_ has also been observed in cancer cells.^[13]^ Neu4,5Ac_2_ is only expressed in certain vertebrates such as monotremes,^[14]^ guinea pigs^[15]^ and horses^[16]^ where it plays roles in disrupting bacterial and viral activity. The disruption has been posited to occur due to steric hindrance in binding sites posed by the protruding 4‐position acetyl group present in Neu4,5Ac_2_.[^14^, ^16^]

Neu5Ac has previously been identified as a biomarker for cancer, diabetes and cardiovascular diseases.^[17]^ Overexpression of sialic acid has been detected on the endothelium when cardiovascular disease (CVD) and therefore inflammation is present. There is a significant elevation in sialic acid in plasma and serum samples between healthy controls and disease patients. Sialic acid can be correlated with CVD risk, with volunteers that exhibit elevated sialic acid levels having higher mortality risk from CVD in a large‐scale, long‐term, follow‐up study.^[18]^ Neu5,9Ac_2_ has also emerged as a potential marker for malignant tumours due to overexpression on the tumour surface.^[13]^ The wide range of biologically available neuraminic acids and the array of biological roles they play, especially in immune response and infection, highlights the need for access to a broad range of sialic acid standards in order to probe their potential utility as biomarkers in a number of therapeutic areas.

Determining whether acetylated sialic acid derivatives are potential biomarkers for a therapeutic area of interest requires a high degree of method stability. Moreover, the reproducibility and reliability of such techniques would benefit from quantitative standards to measure the absolute quantitation of sialic acids in biological fluids. Neu5Ac and Neu5Gc are commercially available, but this is not the case for acetylated sialic acid derivatives. Currently the acetylated forms are derived from biological sources by performing acid release on readily available proteins such as bovine submaxillary mucin which has relatively high levels of a variety of sialic acid types.^[19]^ The acid release of these sialic acids using 0.5 M formic acid and subsequent purification under basic conditions can cause challenges due to acetyl group migration with the former, and hydrolysis with the latter, conditions. This method also only results in a mixture of sialic acids that can be difficult to separate on a large scale due to the similarity in the polarities of the sialic acid derivatives. These mixtures can be useful for qualitative analysis and potential assignment of peaks. Individual standards are required, however, for more accurate assignment and quantitative analysis of sialic acids in biological samples.

**Figure 2:**
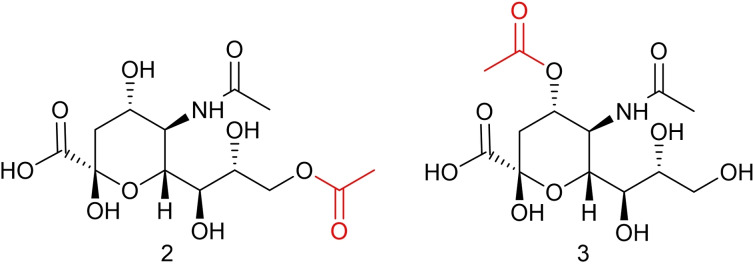
Neu5,9Ac_2_ **(2)** and Neu4,5Ac_2_ **(3)** with acetyl groups highlighted in red.

To address the need for pure, well‐characterised quantitative standards we report herein the further development and optimisation of previously reported synthetic routes towards two quantitative standards of acetylated sialic acid: Neu5,9Ac_2_ and Neu4,5Ac_2_ (Figure 2).^[20]^ Then, to probe the utility of these synthetic derivatives as effective quantitative standards, quantitative nuclear magnetic resonance ( qNMR) techniques were completed to determine the purity of the synthesised compounds. Finally, the synthetic compounds were used as qualitative and quantitative standards for the analysis of sialic acids in biological samples derived from both humans and other species.

## Results and Discussion

### Synthesis of Neu5,9Ac_2_ and Neu4,5Ac_2_

**Scheme 1:**
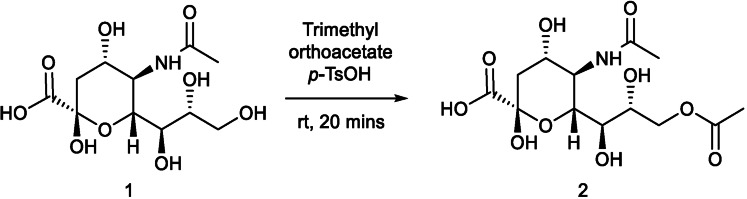
Synthesis of Neu5,9Ac_2_.

Neu5,9Ac_2_ and Neu4,5Ac_2_ have both been previously synthesised by Ogura *et al*.^[20]^ and Clarke *et al*.^[21]^ Neu5,9Ac_2_ was synthesised in one step from Neu5Ac, and Neu4,5Ac_2_ was synthesised in five steps from Neu5Ac by Ogura *et al*. and Clarke *et al*. albeit utilising different protecting group strategies. These approaches therefore lay the foundation for our approach. For access to Neu5,9Ac_2_, Neu5Ac **(1)** was treated with trimethyl orthoacetate in the presence of catalytic *p*‐toluene sulfonic acid for 20 minutes to afford Neu5,9Ac_2_ **(2)** in 68 % yield after purification using ion‐exchange chromatography (Scheme 1). Acetylation at the 9‐position could be confirmed by ^1^H NMR spectroscopic analysis by the presence of a singlet at 2.00 ppm and the shift of the protons at the C‐9‐position hydrogens from 3.46 and 3.68 ppm in **(1)** to 3.95 and 4.26 ppm in **(2)**.

**Scheme 2:**
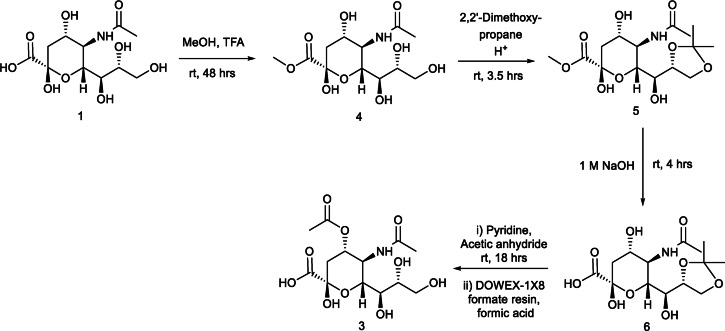
Synthesis of Neu4,5Ac_2_.

The route chosen to synthesise Neu4,5Ac_2_ **(3)** was based on work by Ogura *et al*.^[20]^ The synthesis of **(3)** therefore commenced with protection of the carboxylic acid group to give a methyl ester using conditions developed by Malapelle *et al*. employing methanol and trifluoroacetic acid to give **(4)** in quantitative yield.^[22]^ This was then followed by the simultaneous protection of the 8 and 9‐position hydroxyl groups with an acetonide protecting group using 2,2’‐dimethoxypropane and Amberlyst 15 H^+^ resin to give **(5)** in 65 % yield. Deprotection of the methyl ester is required in the next step. The method put forth by Ogura *et al*. using 1 M NaOH was utilised to give **(6)** in quantitative yield. The acetyl group was installed at the 4‐position using excess pyridine and acetic anhydride and the crude mixture was then purified using ion exchange chromatography using 1 M formic acid as the eluent. Removal of the formic acid under reduced pressure at 35 °C, as opposed to the use of lyophilisation by Ogura *et al*. then afforded **(3)** in 60 % yield (Scheme 2). Pleasingly, it was therefore evident that removal of formic acid under these conditions also facilitated removal of the acetonide protecting group, as required, without the need for an additional synthetic step. As such, the approach detailed herein offers a higher overall yield compared with previous reports, specifically of a 39 % overall yield versus 25 % reported by Ogura *et al*. The successful acetylation at the 4‐positon could be observed via ^1^H NMR spectroscopic analysis by the appearance of a singlet at 1.95 ppm from the new acetyl group protons and the shift of the proton at C‐4 from 3.95 in **(1)** to 5.17 ppm in the target compound **(3)**.

### Quantitative NMR of standards

With Neu5,9Ac_2_ and Neu4,5Ac_2_ in hand, the next step was to determine the purity of the standards using qNMR techniques. The method chosen for this was based on the pulse length based concentration determination (PULCON) method,[^23^, ^24^, ^25^] which utilises an external standard with a known concentration to calculate the concentration of an unknown sample. The PULCON method was chosen, as opposed to a method using an internal standard such as electronic reference to access *in vivo* concentrations (ERETIC),^[26]^ to avoid contamination of the sample. This was due to the limited quantities of material that were available and hence to facilitate further utilisation of the material after qNMR analysis. Analysis was performed by measuring the 360 ° pulse and comparing the integration of a known peak for both the reference and the unknown sample. The measurement of each spectrum was carried out with water suppression using presaturation to ensure that water in the sample would not obscure any peaks and that the maximum peak intensity was obtained. Careful choice of the relaxation delay (d1) is also important; it is crucial for an accurate quantitation to ensure that all signals have fully relaxed between pulses. A d1 of at least five times the T1 of the slowest relaxing signal of interest in the spectrum should be used. T1 values for ^1^H nuclei in medium‐sized molecules typically range from 0.5 to 4.0 seconds.

In our case, maleic acid was used as external standard at a concentration of 5.03 mmol. Maleic acid was chosen as its NMR resonance does not overlap with that of the synthesised standards and is not affected by water suppression. The 360 ° pulse for both standards and samples was calculated using pulsecal; the pulse sequence used was noesypr1D with water suppression using presaturation, and the relaxation time chosen was 20 s.

Neu5Ac was first analysed to validate the method and hence five different concentrations were analysed via qNMR spectroscopy. The signals of interest (the 3‐H, 12‐H, 7‐H and 9‐H protons) in the NMR spectra obtained were integrated and normalised for the number of protons. These signals were chosen as they are distinct, and not overlapped by other resonances. They are also outside of the 3.8–5.8 ppm range and hence would not be inadvertently affected during water suppression. Signals were then compared with the signal of the maleic acid using the equation below (1) where S is the absolute area of the NMR signal, n the number of protons, U is the unknown sample, R is the reference standard and c is concentration.

(1) $$c_{U}=c_{R}\frac{S_{U}n_{R}}{S_{R}n_{U}}$$

The standard curve results were compared with the expected concentrations for each sample (Table 1) which showed that the determined values matched closely to the expected values, indicating the robustness of the PULCON method for concentration determination of a sample. A standard calibration curve is provided in the supporting information.

**Table 1:** Concentration of Neu5Ac standards determined by qNMR compared to the expected concentration of each standard.

Standard number	Prepared concentration of standard [mM]	Concentration determined by qNMR [mM]
1	2.7	2.7
2	4.1	4.2
3	5.4	5.7
4	6.7	7.0
5	8.1	7.8

Having demonstrated the robustness of this qNMR PULCON method, the two synthesised sialic acid standards were next each analysed in triplicate. For Neu5,9Ac_2_ the 3‐H, 7‐H, 12‐H and 15‐H protons were selected for study. For Neu4,5Ac_2_ the 3‐H, 7‐H, 9‐H and 12‐H protons were selected for study. This procedure allowed for the concentrations of the Neu5,9Ac_2_ and Neu4,5Ac_2_ in the standard solutions to be determined, again using equation (1), as 4.04 mM and 3.71 mM, respectively.

### Utilisation of standards for quantitation

**Scheme 3:**
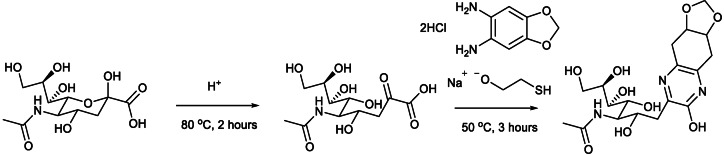
Sialic acid release and DMB labelling reaction.

Standards of 1 nmol of Neu5,9Ac_2_ and Neu4,5Ac_2_ were next dispensed using a liquid handling robot to minimise the introduction of errors. Commercially available 1 nmol Neu5Ac and Neu5Gc provided by Ludger Ltd. were also utilised. Neu5Ac and Neu5Gc have previously been quantified using DMB assays[^27^, ^28^] and provide a good point of comparison for our standards. The standards were labelled with 1,2‐diamino‐4,5‐methyleneoxybenzene (DMB) in the presence of *β*‐mercaptoethanol and sodium dithionite in 1.4 M Acetic Acid (Scheme 3, step 2) and analysed via ultra‐high performance liquid chromatography (UHPLC).[^27^, ^29^] This method was chosen as it is highly specific for sialic acids and allows for the detection of low levels of these carbohydrates. UHPLC also does not suffer from many of the issues that hinder other assays for the quantitative analysis of sialic acids, for example that other compounds present in complex biological samples can affect the quantitation procedure by reacting with the reagents used.^[30]^

**Figure 3:**
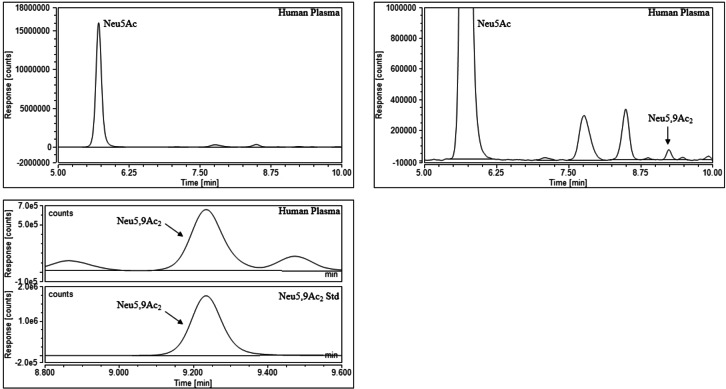
Human plasma after DMB labelling and UHPLC analysis (full trace and zoomed in).

**Figure 4:**
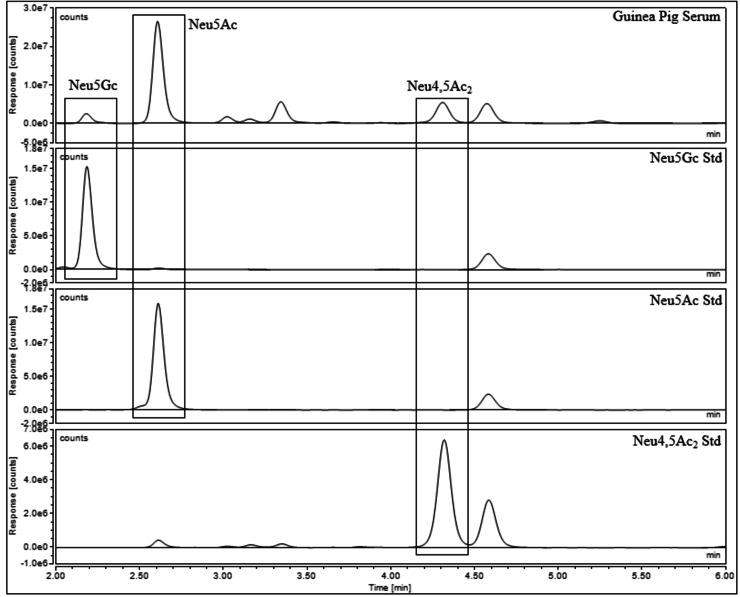
Guinea pig serum after DMB labelling and UHPLC analysis.

**Figure 5:**
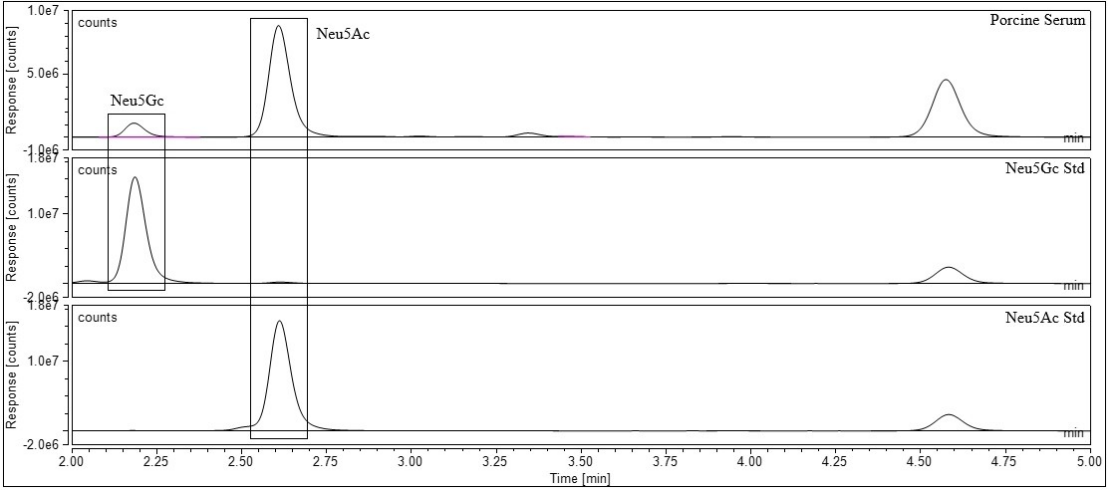
Porcine serum after DMB labelling and UHPLC analysis.

**Figure 6:**
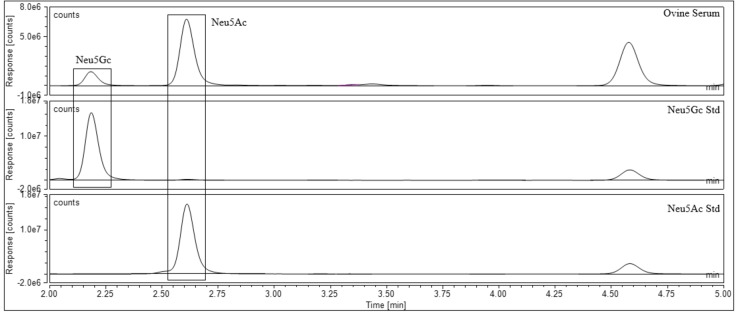
Ovine serum after DMB labelling and UHPLC analysis.

Once the standards were qualitatively assessed, the next stage was to utilise the standards for the quantitation of sialic acids in biological samples. Samples that contained acetylated sialic acid derivatives were chosen to validate Neu5,9Ac_2_ and Neu4,5Ac_2_ as quantitative standards. A literature search of biologically available Neu5Ac derivatives led to the purchase of recombinant human plasma^[31]^ and guinea pig serum^[15]^ from Sigma‐Aldrich, and dried porcine and ovine serum[^1^, ^3^] from First Link (UK). The sialic acids were released from the biological samples using 2 M acetic acid at 80 °C for 2 hours; these conditions are kept intentionally mild to ensure that no acetyl group migration takes place (Scheme 3, step 1). Following this, both standards and samples were labelled with DMB at 50 °C for 3 hours (Scheme 3, step 2). The standards were used to qualitatively assess which sialic acids were present in each sample and to create standard curves for quantitative analysis. A variety of sialic acids were detected and quantified in each sample. Neu5Ac and Neu5,9Ac_2_ were detected in human plasma (Figure 3). Neu5Gc, Neu5Ac and Neu4,5Ac_2_ were detected in guinea pig serum (Figure 4), and this was the only sample to contain Neu4,5Ac_2_. Porcine and ovine serum showed Neu5Ac and Neu5Gc (Figures 5 and 6). The information obtained matched the expectations derived from previous literature; Neu5Ac and Neu5,9Ac_2_ are the main neuraminic acid derivatives identified in humans.[^32^, ^33^] The animal serum samples also match previous literature, as all three samples contained Neu5Gc, which is not present in human samples.^[34]^ Neu4,5Ac_2_ was detected in guinea pig serum.^[15]^ This method therefore allowed for the identification of a variety of different sialic acid derivatives in complex biological mixtures with the need for only one assay per sample, greatly reducing the time required to perform analysis of these compounds. Other unknown peaks were also observed in the traces obtained for the animal samples, but their identification was outside of the scope of this analysis, which was to determine the validity of the synthesised compounds as quantitative standards.

Neu5Ac, Neu5Gc and Neu4,5Ac_2_ could be effectively quantified in small quantities of the relevant biological samples with ease: the peaks met the thresholds for limit of detection (LOD) and limit of quantitation (LOQ). Neu5,9Ac_2_ posed an issue in the plasma sample in that it was present in quantities 50‐100 times smaller than Neu5Ac and as such the samples were concentrated before analysis to ensure that they met the thresholds of the LOD and LOQ (Table 2). This sample also suffered from impurities overlapping the Neu5,9Ac_2_ peak. The lack of sufficient resolution was overcome by changing the solvent system to effectively separate Neu5,9Ac_2_ from any impurities ‐ using a gradient solvent system starting with low acetonitrile content and slowly increasing the volume to give a greater retention time for sialic acid derivatives allowed for any impurities to elute first. The values obtained for quantitative analysis (Tables 3 and 4) were as expected: Neu5,9Ac_2_ is generally detected in quantities far smaller than that of Neu5Ac.[^32^, ^33^] Neu4,5Ac_2_ was found to comprise 34 % of total sialic acid in guinea pig serum, which matched the previously reported data for this material.^[15]^

**Table 2:** Limit of detection and limit of quantitation of Neu5Ac, Neu5Gc, Neu5,9Ac_2_ and Neu4,5Ac_2_.

Standard	LOD [nmol]	LOD [ng]	LOQ [nmol]	LOQ [ng]	R^2^ std curve
Neu5Ac	0.0100	3.09	0.0305	9.42	1.00
Neu5Gc	0.0085	2.76	0.0257	8.35	1.00
Neu5,9Ac_2_	0.0059	2.07	0.0180	6.32	1.00
Neu4,5Ac_2_	0.0108	3.79	0.0329	11.54	1.00

**Table 3:** Quantities of different different sialic acids in biological samples. The data are presented as mean ± standard deviation.

Sample	Average Neu5Ac [mg/100 mL]	Average Neu5Gc [mg/100 mL]	Average Neu5,9Ac_2_ [mg/100 mL]	Average Neu4,5Ac_2_ [mg/100 mL]
Human plasma	45.49±0.99	N/A	0.29±0.01	N/A
Guinea pig serum	37.80±1.15	4.21±0.10	0.33±0.04	21.25±0.40

**Table 4:** Quantity different sialic acids in biological samples. The data are presented as mean ± standard deviation.

Sample	Average Neu5Ac [mg/100 mg serum]	Average Neu5Gc [mg/100 mg serum]	Average Neu5,9Ac_2_ [mg/100 mg serum]	Average Neu4,5Ac_2_ [mg/100 mg serum]
Porcine serum	0.69±0.092	0.10±0.012	N/A	N/A
Ovine serum	0.44±0.038	0.11±0.0092	N/A	N/A

The LOD and LOQ found for Neu5Ac were in line with previous literature^[27]^ with the method outlined here boasting a similar LOD and LOQ for Neu5Gc, Neu5,9Ac_2_ and Neu4,5Ac_2_. The intra‐assay variation for the samples in triplicate was <10 % indicating that the assay has good precision and allows for repeatable results while offering the ability to identify and quantify multiple sialic acid derivatives in the same assay.

## Conclusions

The synthesis of 4‐*O*‐ and 9‐*O*‐acetylated sialic acids (Neu5,9Ac_2_ and Neu4,5Ac_2_) was achieved through optimisation of previously reported synthetic routes. Neu5,9Ac_2_ was synthesised in 1 step in 68 % yield. Neu4,5Ac_2_ was synthesised in 4 steps in 39 % overall yield. The analysis of these standards by qNMR revealed the concentration of each standard present in the prepared samples and gave an accurate determination of the quantity of sample to dispense that could then be utilised as qualitative and quantitative standards for the analysis of biological samples. Methods were developed for the utilisation of these standards for the quantitation of Neu5,9Ac_2_ and Neu4,5Ac_2._ The method enabled the separation, detection and quantification of multiple derivatives of sialic acid simultaneously, forgoing the need for multiple assays to detect different derivatives in the same sample. The method was also highly sensitive, exhibiting a limit of detection and limit of quantitation comparable with previously reported DMB assays for Neu5Ac, thereby indicating that these standards combined with the DMB labelling method employed here can be used to analyse acetylated sialic acid derivatives even when present in small (<0.1 nmol) quantities without interference from impurities. The ability to detect and quantify different derivatives of sialic acid in biological samples opens doors for the investigation of these derivatives as biomarkers for different diseases such as CVD or malignant tumours.

## Experimental Section

**Instrumentation**: Melting points were determined using a Stuart SMP10 melting point apparatus. Optical rotations were recorded using a Perkin Elmer Polarimeter 341 with reference to the sodium D line (λ=589 nm, sodium lamp) and values are given in 10^−1^ deg.cm^2^.g^−1^. Infra‐red spectra were recorded using a Thermo Scientific Nicolet IS5 FT‐IR spectrophotometer with an ID5 ATR accessory onto which a small quantity of sample (10 mg) was placed as a solid and values are given as the wavenumber (cm^−1^). Mass spectrometry was performed by the University of Reading Chemical Analysis Facility using a Thermo Scientific LTQ OrbiTrap XL with an attached ACCELA LC Autosampler. NMR spectra were recorded using either a Nanobay or Bruker DPX 400 spectrometer at 400 MHz for ^1^H and 100 MHz for ^13^C in either DMSO or D_2_O. Quantitative NMR was carried out using a Bruker Advance III 500 MHz instrument in D_2_O. Chemicalshifts are quoted in parts per million. Coupling constants are quoted in Hertz (Hz) and rounded to the nearest 0.5 Hz.

**5‐Acetamido‐3,5‐dideoxy‐9‐*O*‐acetyl‐D‐glycero‐D‐galactononulopyranosonate (2)**:^[20]^ To a solution of *N*‐acetylneuraminic acid **1** (0.25 g, 0.81 mmol, 1 eq.) in DMSO containing *p*‐TsOH (10 mg) was added trimethyl orthoacetate (0.205 μL, 1.62 mmol, 2 eq.). The reaction was stirred at room temperature for 20 minutes. The resultant reaction mixture was poured directly onto a column (2×10 cm) of DOWEX‐1X8 formate anion exchange resin (100‐200 mesh). The column was washed with water (3×10 mL) and eluted with formic acid (50 mL). The formic acid was removed under reduced pressure to give a clear residue. The residue was dissolved in water (5 mL) and lyophilised to give 9‐*O* acetyl *N*‐acetylneuraminic acid **2** as a white solid (175 mg, 68 % yield). [α]20D −13 ° (c 1.0, H_2_O); mp. 153–155 °C; ^1^H NMR (400 MHz, D_2_O): δ 4.26 (1H, dd, *J*=11.5, 2.5 Hz, H‐9), 4.07 (1H, dd, *J*=6.5 Hz, H‐4), 4.00–3.90 (2H, m, H‐6+H‐9), 3.87–3.77 (2H, m, H‐5+H‐8), 3.49 (1H, d, *J*=8.0 Hz, H‐7), 2.19 (1H, dd, *J*=13.0, 5.0 Hz, H‐3eq), 2.00 (s, 3H, H‐15), 1.94 (s, 3H, H‐12), 1.81‐1.72 (1H, m, H‐3ax ^13^C NMR (100 MHz, D_2_O): δ 174.73 (C13), 174.41 (C11), 171.22 (C14), 95.48 (C2), 70.15 (C7), 69.2 (C8), 67.59 (C6), 66.80 (C4), 66.26 (C9), 52.03 (C5), 38.89 (C3), 22.6 (C12), 20.19 (C15) ppm; IR *v*_max_ [cm^−1^] (powder) 3295 (O−H, br), 1719 (C=O), 1034 (C−O); HRMS (ESI): m/z calc for C_13_H_22_O_10_N: 352.1238 [M+H]^+^_;_ found: 351.1239.

**Methyl 5‐Acetamido‐3,5‐dideoxy‐D‐glycero‐D‐galactononulopyranosonate (4)**:^[22]^ To a solution of *N*‐acetylneuraminic acid **1** (2.0 g, 6.46 mmol, 1 eq.) in methanol (40 mL) was added TFA (0.98 mL, 12.92 mmol, 2 eq.). This was stirred at room temperature for 48 hrs. The solvent was removed under reduced pressure to give the methyl ester **4** as a white solid (2.1 g, quant.) [α]20D −26 ° (c 1.0, MeOH); mp. 177–178 °C; ^1^H NMR (400 MHz, D_2_O): δ 3.94 (2H, m, H‐4+H‐6), 3.80 (1H, m, H‐9), 3.72 (4H, m, H‐5+H‐14), 3.60 (1H, m, H‐8), 3.50 (1H, m, H‐9), 3.42 (1H, m, H‐7), 2.19 (1H, dd, H‐3eq *J*=13.0, 5.0 Hz), 1.92 (3H, s, H‐12), 1.79 (1H, m, H‐3ax) ppm ^13^C NMR (100 MHz, D_2_O): δ 174.79 (C13), 171.36 (C11), 95.29 (C2), 70.29 (C6), 70.05 (C8), 68.14 (C7), 66.62 (C4), 63.10 (C9), 53.43 (C14), 52.00 (C5), 38.60 (C3), 22.00 (C12) ppm; IR *v*_max_ [cm^−1^] (powder) 3256 (O−H, br), 2944 (C−H), 1751 (C=O), 1026 (C−O); HRMS (ESI): m/z calc for C_12_H_21_O_9_NNa: 352.1109 [M+H]^+^_;_ found: 346.1102.

**Methyl 5‐acetamido, 3,5‐dideoxy‐8,9‐*O*‐isopropylidene‐D‐glycero‐β‐D‐galactononulopyranosonate (5)**:^[20]^ To a solution of methyl ester **4** (1.0 g, 3.09 mmol, 1 eq.) in acetone (50 mL) was added Amberlyst 15 H^+^ resin (1.5 g) and 2,2‐dimethoxypropane (0.48 mL, 3.71 mmol, 1.2 eq.). This was stirred for 3.5 hrs at room temperature before being filtered to remove the resin. The resin was washed with acetone and the filtrate collected. After removing the solvent under reduced pressure, the resulting crude material was passed over a silica plug and eluted with ethyl acetate (4×200 mL) to give acetonide **5** as a beige foam (0.73 g, 65 % yield) [α]20D −24 ° (c 1.0, MeOH); mp. 164–166 °C; ^1^H NMR (400 MHz, DMSO‐d6): δ 8.07 (1H, d, *J*=8.0 Hz, H‐10), 6.71 (1H, d, *J*= 2.0 Hz, O−H2), 4.82 (1H, d, J=6.0 Hz, O−H4), 4.75 (1H, d, *J*=5.0 Hz, O−H7), 4.02‐3.94 (1H, m, H‐8), 3.90‐3.86 (1H, m, H‐6), 3.85‐3.78 (2H, m, H‐4 +H‐9), 3.68 (3H, s, H‐14), 3.56 (1H, t, *J*=9.5 Hz, H5), 3.52‐3.49 (2H, m, H‐7+H‐9), 2.03 (1H, dd, *J*=13.0, 5.0 Hz, H‐3eq), 1.88 (3H, s, H‐12), 1.61 (1H, m, H‐3ax), 1.26 (3H, s, H16), 1.23 (3H, s, H‐16) ppm ^13^C NMR (100 MHz, DMSO‐d6): δ 179.94 (C13), 171.68 (C11), 107.22 (C15), 94.57 (C2), 75.92 (C8), 71.70 (C7), 68.38 (C6), 65.20 (C9), 64.94 (C4), 52.89 (C14), 52.12 (C5), 40.23 (C3), 26.55 (C16), 25.72 (C16), 22.55 (C12) ppm; IR *v*_max_ [cm^−1^] (powder) 3291 (O−H, br), 2926 (C−H), 1740 (C=O), 1032 (C−O); HRMS (ESI): m/z calc for C_15_H_25_O_9_NNa: 386.1422 [M+Na]^+^_;_ found: 386.1416.

**5‐Acetamido‐3,5‐dideoxy‐8,9‐*O*‐isopropylidene‐D‐glycero‐D‐galactononulopyranosonate (6)**:^[20]^ The acetonide **5** (0.25 g, 0.68 mmol) was dissolved in 1 M NaOH (5 mL) and stirred for 4 hrs at room temperature. This was then diluted with water (10 mL) and deionised with Amberlyst H^+^ resin. The mixture was filtered, the filtrate was collected and lyophilised to give a white solid **6** (0.24 g, quant.). [α]20D −25 ° (c 1.0, H_2_O); mp. 157–158  °C; ^1^H NMR (400 MHz, D_2_O): δ 4.25 (1H, dd, *J*=12.5 Hz, 6.0 Hz, H‐8), 4.20‐4.11 (1H, m, H‐6), 4.07‐3.97 (2H, m, H‐4+H‐9), 3.95‐3.85 (2H, m, H‐5+H‐9), 3.62 (1H, m, *J*=7.5 Hz, H‐7), 2.09 (3H, s, H‐12), 1.46 (3H, s, H‐15), 1.36 (3H, s, H‐15) ppm; ^13^C NMR (100 MHz, D_2_O): δ 176.75 (C13), 174.43 (C11), 168.30 (C2), 109.58 (C14), 75.10 (C8), 70.59 (C7), 69.07 (C6), 66.99 (C9), 66.14 (C4), 52.16 (C5), 25.77 (C15), 24.27 (C15), 21.99 (C12) ppm; IR *v*_max_ [cm^−1^] (powder) 3282 (O−H, br), 1735 (C=O), 1427 (COOH) 1057 (C−O); HRMS (ESI): m/z calc for C_14_H_23_O_9_NNa: 372.1265 [M+H]^+^_;_ found: 372.1263.

**5‐Acetamido‐3,5‐dideoxy‐4‐*O*‐acetyl‐D‐glycero‐D‐galactononulopyranosonate (3)**:^[20]^ To a solution of **6** (100 mg, 0.29 mmol, 1 eq.) in dry pyridine (0.5 mL) under nitrogen was added acetic anhydride (30 μL, 0.32 mmol, 1.1 eq.). This was stirred at room temperature for 18 hours after which ethanol was added to remove excess acetic anhydride and solvent removed under reduced pressure by co‐evaporation with toluene (3×100 mL). The resultant residue was dissolved in water (10 mL) and passed through a column (2×10 cm) of DOWEX‐1X8 formate anion exchange resin (100–200 mesh). The column was washed with water (3×10 mL) and eluted with formic acid (50 mL). The formic acid was removed under reduced pressure to give a clear residue. The residue was dissolved in water (5 mL) and lyophilised to give 4‐*O N*‐acetylneuraminic acid **3** as a white solid (60 mg, 60 % yield). [α]20D −34° (c 1.0, H_2_O); mp. 170–172 °C; ^1^H NMR (400 MHz, D_2_O): 5.22‐5.12 (1H, m, H‐4), 4.18‐3.99 (2H, m, H‐5+H‐6), 3.76‐ 3.72 (1H, m, H‐9), 3.63 (1H, m, H‐8), 3.50 (1H, m, H‐9+H‐7), 2.24 (1H, m, H‐3eq), 1.95 (3H, s, H‐15), 1.87 (4H, m, H‐12+H‐3ax); ^13^C NMR (100 MHz, D_2_O): δ 174.7 (C14), 173.38 (C13), 173.31 (C11), 95.24 (C2), 70.07 (C8), 70.00 (C7), 68.01 (C6), 63.09 (C4), 53.50 (C9), 49.38 (C5), 36.02 (C3), 21.85 (C12), 20.30 (C15) ppm; IR *v*_max_ [cm^−1^] (powder) 3340 (O−H, br), 2933 (C−H), 1727 (C=O), 1019 (C−O); HRMS (ESI): m/z calc for C_13_H_22_O_10_N: 352.1238 [M+H]^+^_;_ found: 351.1239.

**Quantitative NMR analysis**: Into an NMR tube was placed 600 μL of a 5.03 mMol maleic acid solution in D_2_O. A 1.8 mg quantity of synthesised standard was dissolved in 1.8 mL of D_2_O. This was split into three 600 μL samples in order to analyse the standards in triplicate. After matching and tuning, each NMR sample was analysed on a 500 MHz NMR spectrometer where a 1D ^1^H spectrum with water suppression was obtained with a relaxation delay of 20 seconds. The 360 ° pulse was obtained for each spectrum using pulsecal. The pulse sequence used was noesypr1D. Equation 1 was used to determine the concentration and purity of each synthesised standard.

**DMB labelling of the Neu5Ac, Neu5Gc, Neu5,9Ac_2_ and Neu4,5Ac_2_ standards**: Neu5Ac, Neu5Gc, Neu5,9Ac_2_ and Neu4,5Ac_2_ standards were labelled using LudgerTag^TM^ DMB Sialic Acid (LT‐KDMB‐96). DMB labelling solution (20 μL) was added to the standards. The samples were then vortexed and centrifuged followed by incubation at 50 °C for 3 hours. The labelling reaction was quenched by the addition of water to give a final volume of 500 μL (480 μL). Standard curves were prepared by performing serial dilution to create a standard curve with points: 0.01, 0.02, 0.1, 0.2, 0.5, 1.0 nmol. The procedure was carried out using a Hamilton MICROLAB STARlet Liquid Handling Robot.

**Sialic acid release and DMB labelling of human plasma and guinea pig serum**: Release of sialic acid and DMB labelling of the samples was achieved using LudgerTag^TM^ DMB Sialic Acid (LT‐KDMB‐96). A 5 μL aliquot of each sample was dispensed into a 96‐well plate in triplicate. To this was added 25 μL of 2 M acetic acid. The sample was vortexed and centrifuged followed by incubation at 80 °C for 2 hours. The sample was allowed to cool to room temperature and a 5 μL aliquot of each sample was transferred to a new 96‐well plate. To this was added 20 μL of DMB labelling solution. The sample was then vortexed and centrifuged followed by incubation at 50 °C for 3 hours. The labelling reaction was quenched by the addition of water to give a final volume of 500 μL (475 μL). The samples were then subjected to a 1 in 10 dilution (50 μL sample, 450 μL water). All procedures, except the dispensing of the samples on the plate, were conducted using a Hamilton MICROLAB STARlet Liquid Handling Robot.

**Sialic acid release and DMB labelling of porcine and ovine serum**: Release of sialic acid and DMB labelling of the samples was achieved using LudgerTag^TM^ DMB Sialic Acid (LT‐KDMB‐96). A stock solution was first prepared by dissolving 10 mg of porcine or ovine serum in 1 mL ultra‐pure water. A 10 μL aliquot taken from the solution of each sample was dispensed into a 96‐well plate in triplicate. To this was added 25 μL of 2 M acetic acid. The sample was vortexed and centrifuged followed by incubation at 80 °C for 2 hours. The sample was allowed to cool to room temperature and a 5 μL aliquot of each sample was transferred to a new 96‐well plate. To this was added 20 μL of DMB labelling solution. The sample was then vortexed and centrifuged followed by incubation at 50 °C for 3 hours. The labelling reaction was quenched by the addition of water to give a final volume of 500 μL (475 μL). The samples were then subjected to a 1 in 10 dilution (50 μL sample, 450 μL water). All procedures, except the dispensing of the samples on the plate, were carried out using a Hamilton MICROLAB STARlet Liquid Handling Robot.

**Sialic acid release and DMB labelling of plasma for Neu5,9Ac_2_**: Release of Sialic Acid and DMB labelling of the samples was achieved using LudgerTag^TM^ DMB Sialic Acid (LT‐KDMB‐96). A 25 μL aliquot of each sample was dispensed into a 96‐well plate in triplicate. To this was added 75 μL of 2.666 M acetic acid. The sample was vortexed and centrifuged followed by incubation at 80 °C for 2 hours. The sample was allowed to cool‐room temperature and a 20 μL aliquot of each sample was transferred to a new 96‐well plate. To this was added 20 μL of DMB labelling solution. The sample was then vortexed and centrifuged followed by incubation at 50 °C for 3 hours. The labelling reaction was quenched by the addition of water to give a final volume of 500 μL (475 μL). The sample was subjected to filtration through a Ludger Clean Protein Binding Membrane filtration plate (LC‐PBM‐plate) to remove excess protein. All procedures, except the dispensing of the samples on the plate and LC‐PBM‐plate filtration, were carried out using a Hamilton MICROLAB STARlet Liquid Handling Robot.

**Fluorescence analysis of DMB labelled sialic acid derivatives by LC‐fluorescence detection**: DMB labelled sialic acid derivatives were analysed by LC‐FLD. A 5 μL aliquot of each sample was injected to a LudgerSep™ uR2 UHPLC column (2.1 × 150 mm, 1.9 μm particle size silica derivatized with octadecylsiliane coating, 175 Å pore size) at 30 °C on a Dionex UltiMate™3000 RSLCnano system with a fluorescent detector (λ_ex_ = 373 nm, λ_em_ = 448 nm). For Neu5Ac analysis, an isocratic solvent system was used (7 : 9 : 84 MeOH : ACN : H_2_O) for 15 minutes including an ACN wash. For Neu5,9Ac_2_ analysis a variable solvent system was used: 7 : 6 : 87 MeOH:ACN:H2O for 6.5 minutes followed by 6 : 9 : 85 MeOH:ACN:H2O for 11.5 minutes. Integration of resultant peaks was performed using Chromeleon 7. LOD and LOQ regression analysis and calculation was performed using Microsoft Excel 2019.

1

## Supporting information

As a service to our authors and readers, this journal provides supporting information supplied by the authors. Such materials are peer reviewed and may be re‐organized for online delivery, but are not copy‐edited or typeset. Technical support issues arising from supporting information (other than missing files) should be addressed to the authors.

Supporting Information

## Data Availability

The data that support the findings of this study are available in the supplementary material of this article.
