# Supplementary material for: Quantitative Standards of 4‐O‐Acetyl‐ and 9‐O‐Acetyl‐N‐Acetylneuraminic Acid for the Analysis of Plasma and Serum
Source: Chembiochem. 2022 Jan 18;23(5):e202100662. doi: 10.1002/cbic.202100662 (PMC9303589; doi:10.1002/cbic.202100662)
Supplement: Supplementary file 1 — Supporting Information [file CBIC-23-0-s001.pdf]

ChemBioChem

Supporting Information

Quantitative Standards of 4-*O*-Acetyl- and 9-*O*-Acetyl-*N*-Acetylneuraminic Acid for the Analysis of Plasma and Serum

Jack Cheeseman, Concepcion Badia, Rebecca I. Thomson, Gunter Kuhnle, Richard A. Gardner, Daniel I. R. Spencer, and Helen M. I. Osborn*

NMR Spectra

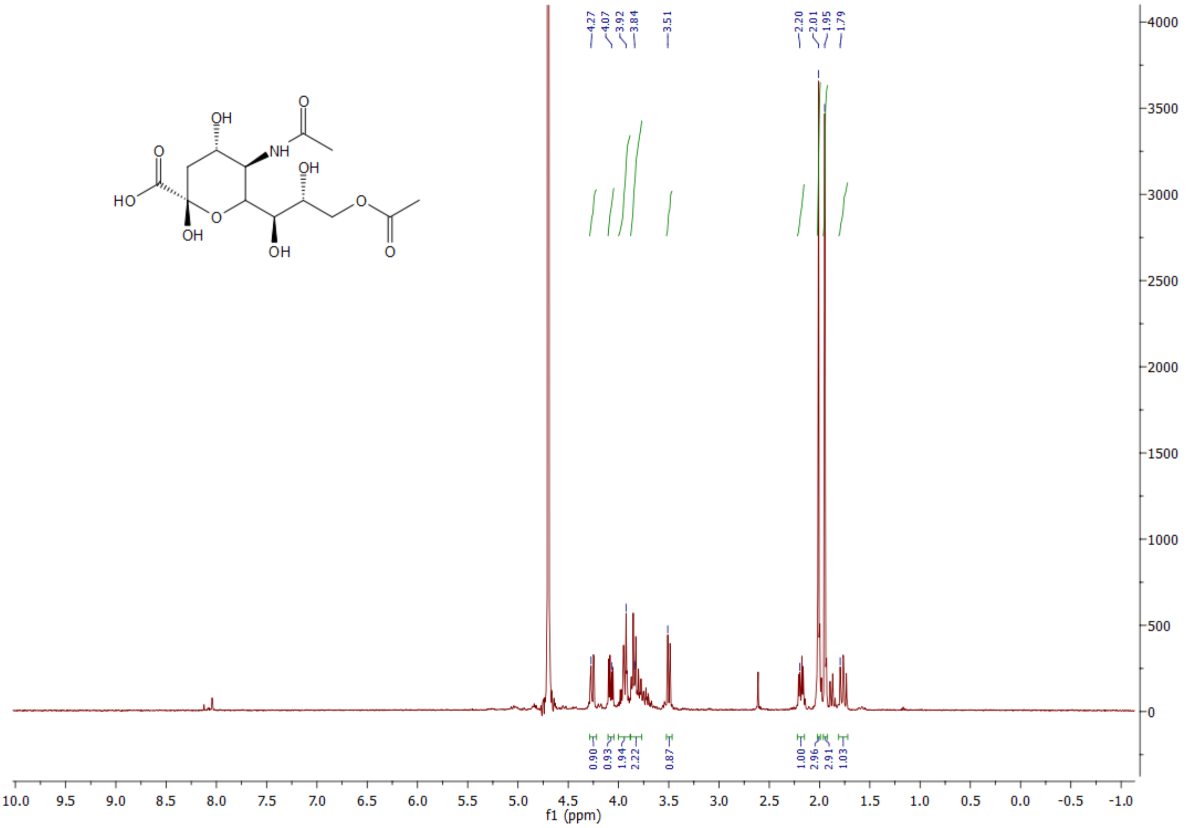

^{1}H NMR (400 MHz, D_2O): 5-Acetamido-3,5-dideoxy-9-*O*-acetyl-D-glycero-D-galactononulopyranosonate **(2)**

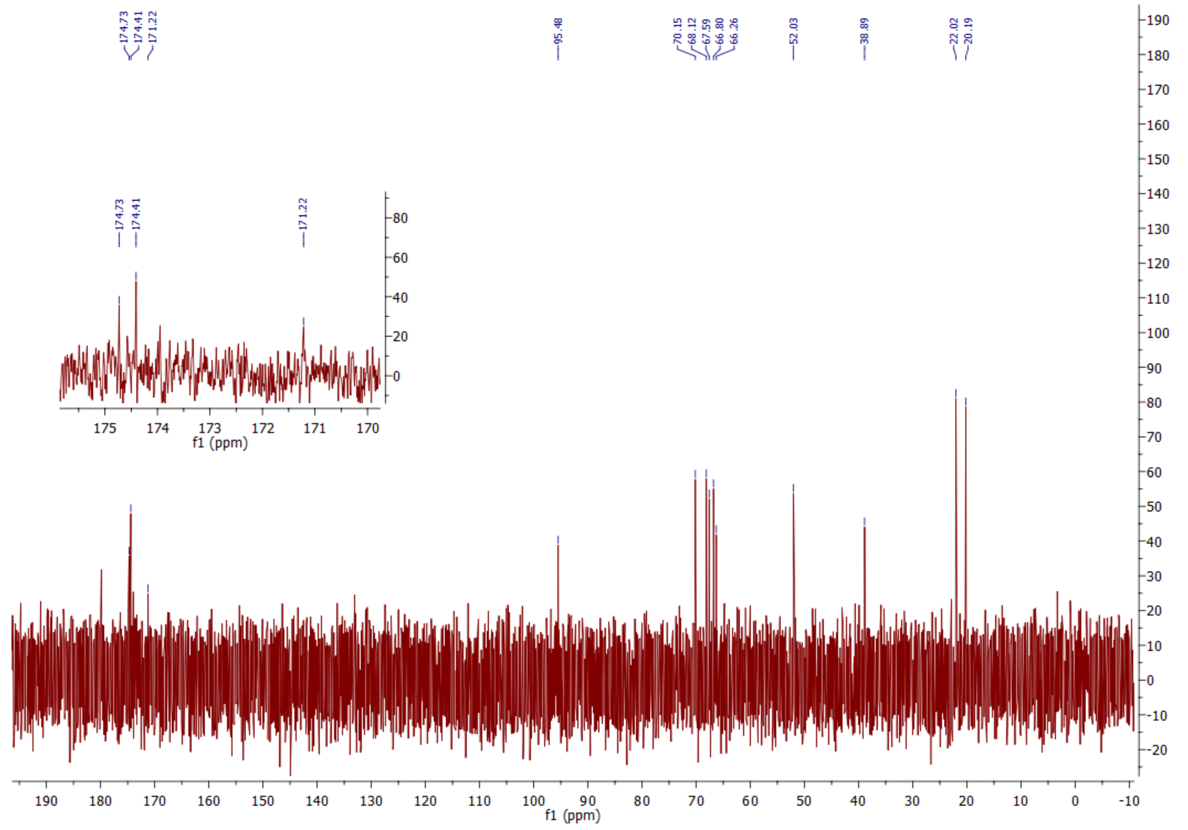

^{13}C NMR (100 MHz, D_2O): 5-Acetamido-3,5-dideoxy-9-*O*-acetyl-D-glycero-D-galactononulopyranosonate **(2)**

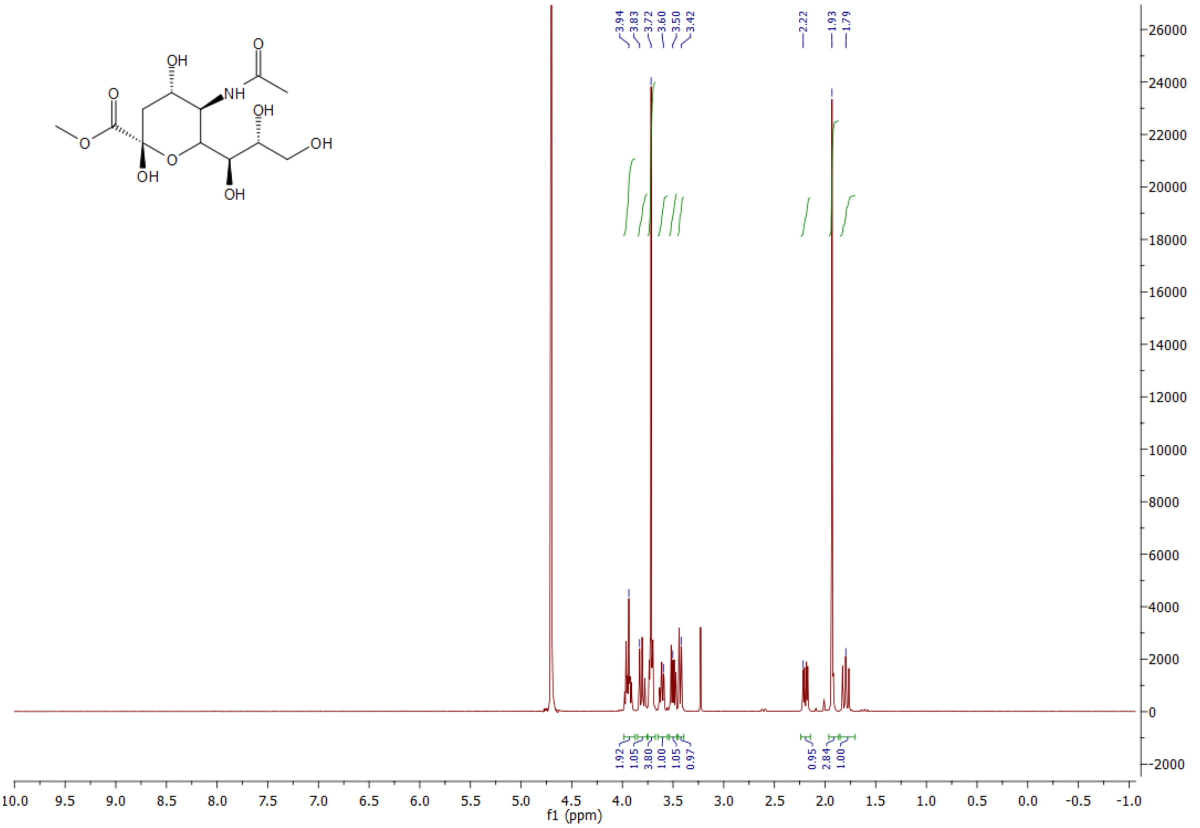

^{1}H NMR (400 MHz, D_2O): Methyl 5-Acetamido-3,5-dideoxy-D-glycero-D-galactononulopyranosonate **(4)**

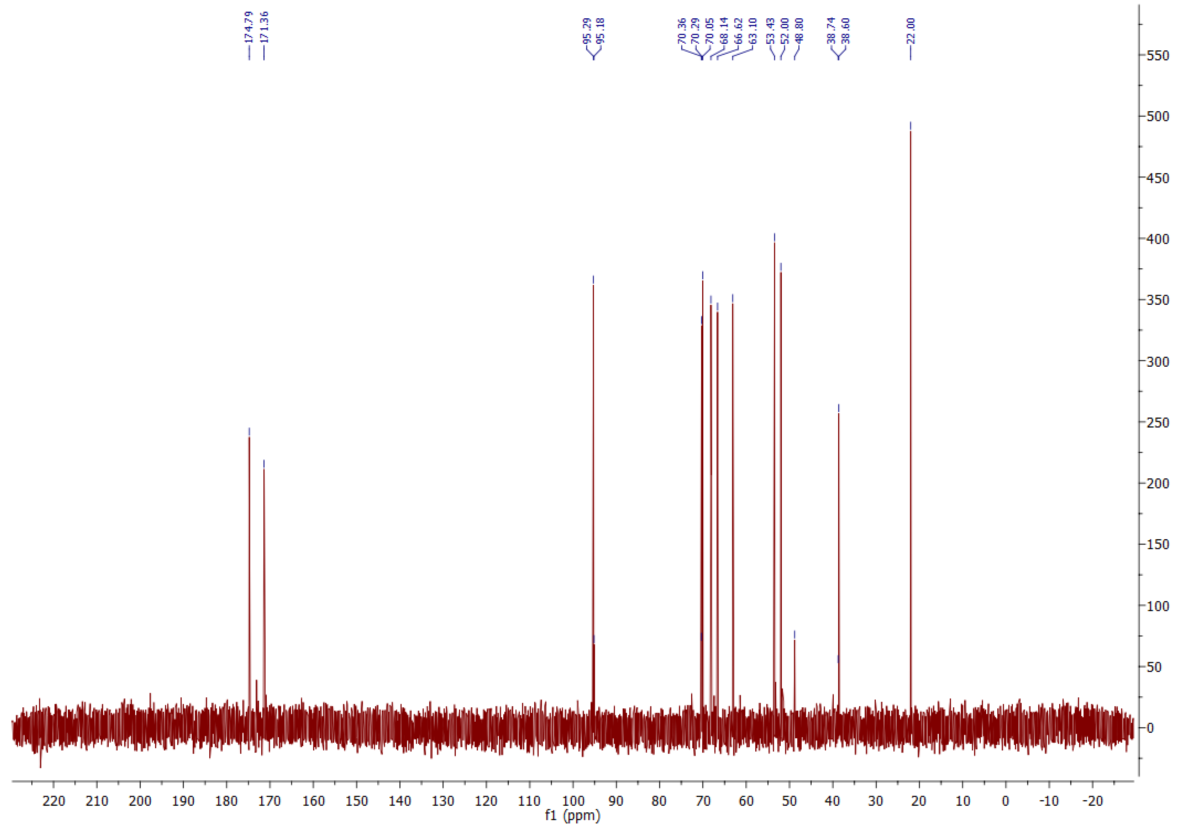

^{13}C NMR (100 MHz, D_2O): Methyl 5-Acetamido-3,5-dideoxy-D-glycero-D-galactononulopyranosonate **(4)**

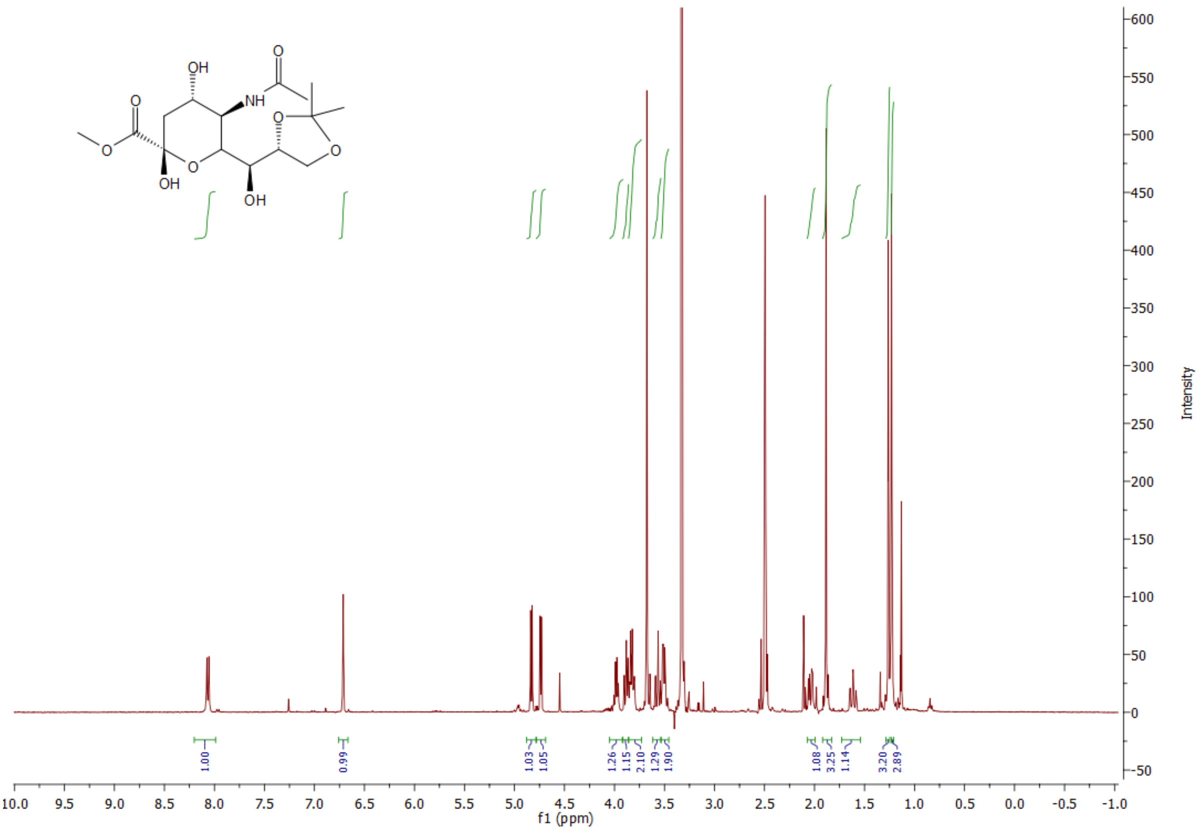

^{1}H NMR (400 MHz, DMSO-d6): Methyl 5-acetamido, 3,5-dideoxy-8,9-*O*-isopropylidene-D-glycero-β-D-galactononulopyranosonate **(5)**

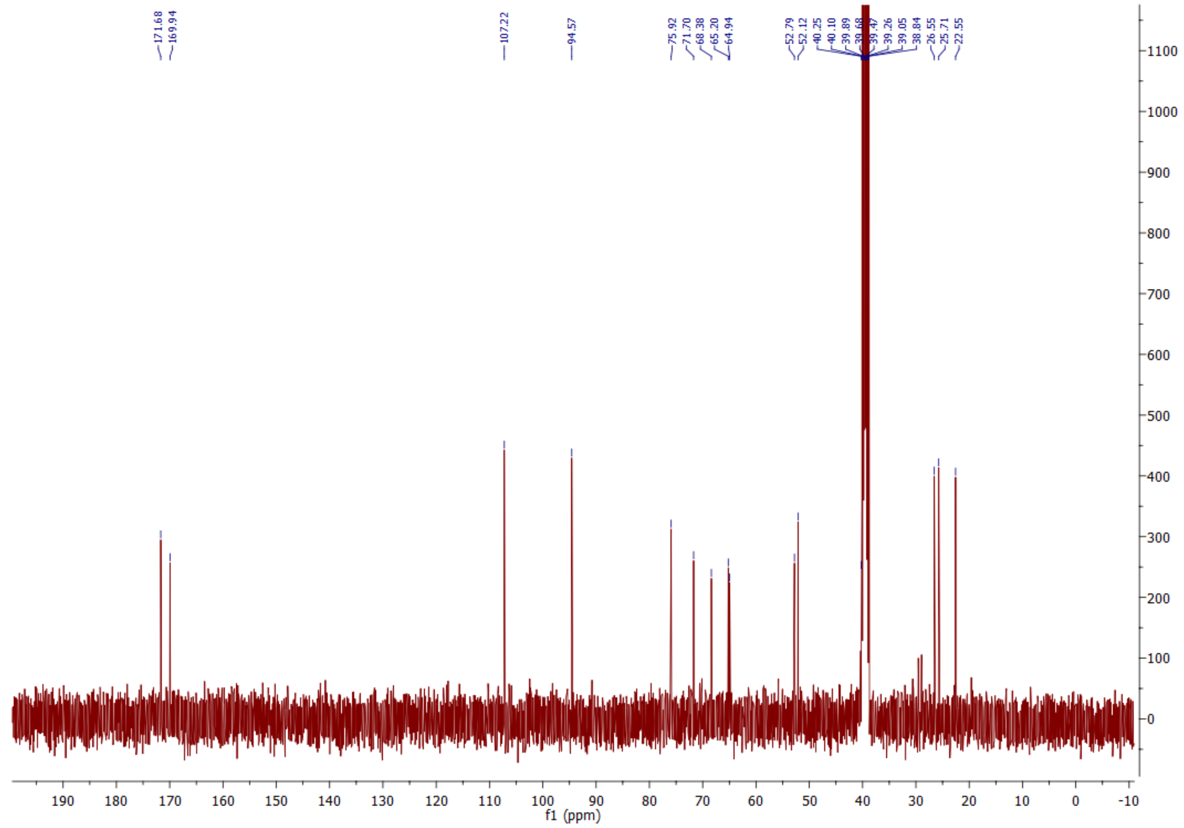

^{13}C NMR (100 MHz, DMSO-d6): Methyl 5-acetamido, 3,5-dideoxy-8,9-*O*-isopropylidene-D-glycero-β-D-galactononulopyranosonate **(5)**

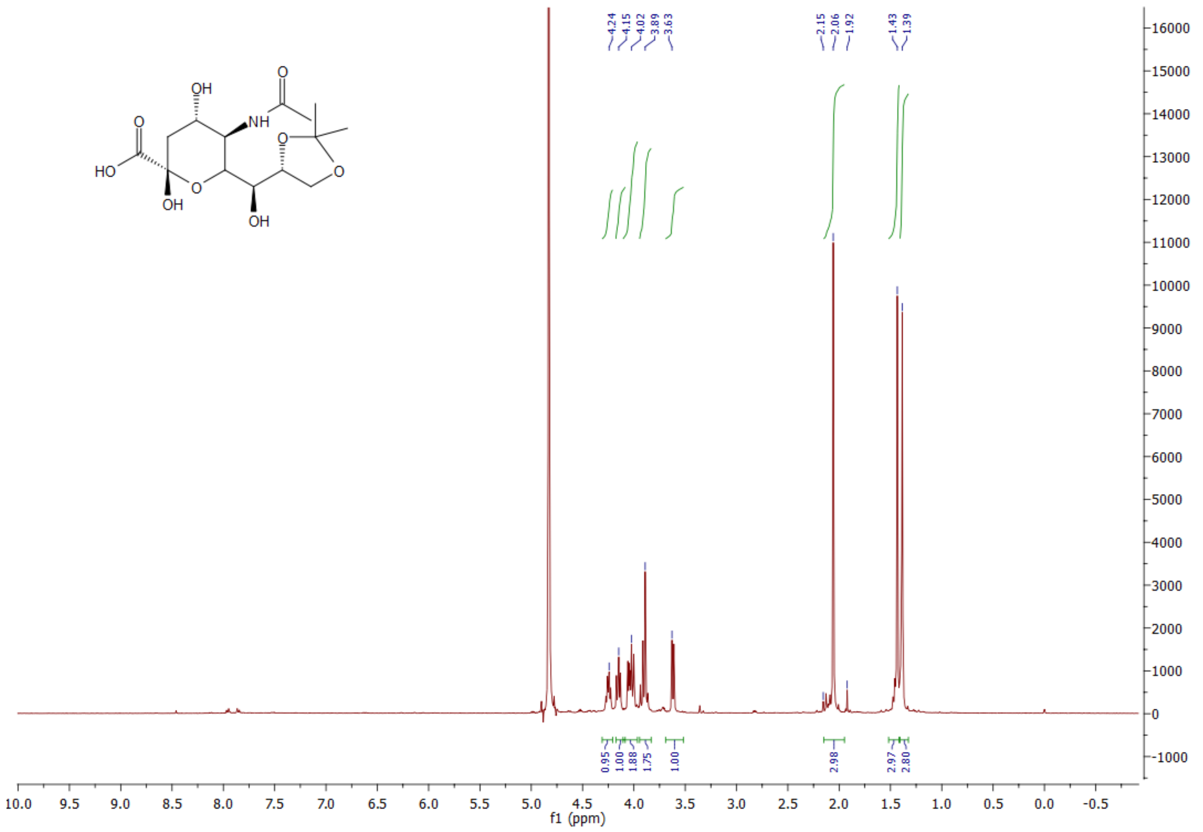

^{1}H NMR (400 MHz, D_2O): 5-Acetamido-3,5-dideoxy-8,9-*O*-isopropylidene-D-glycero-D-galactononulopyranosonate **(6)**

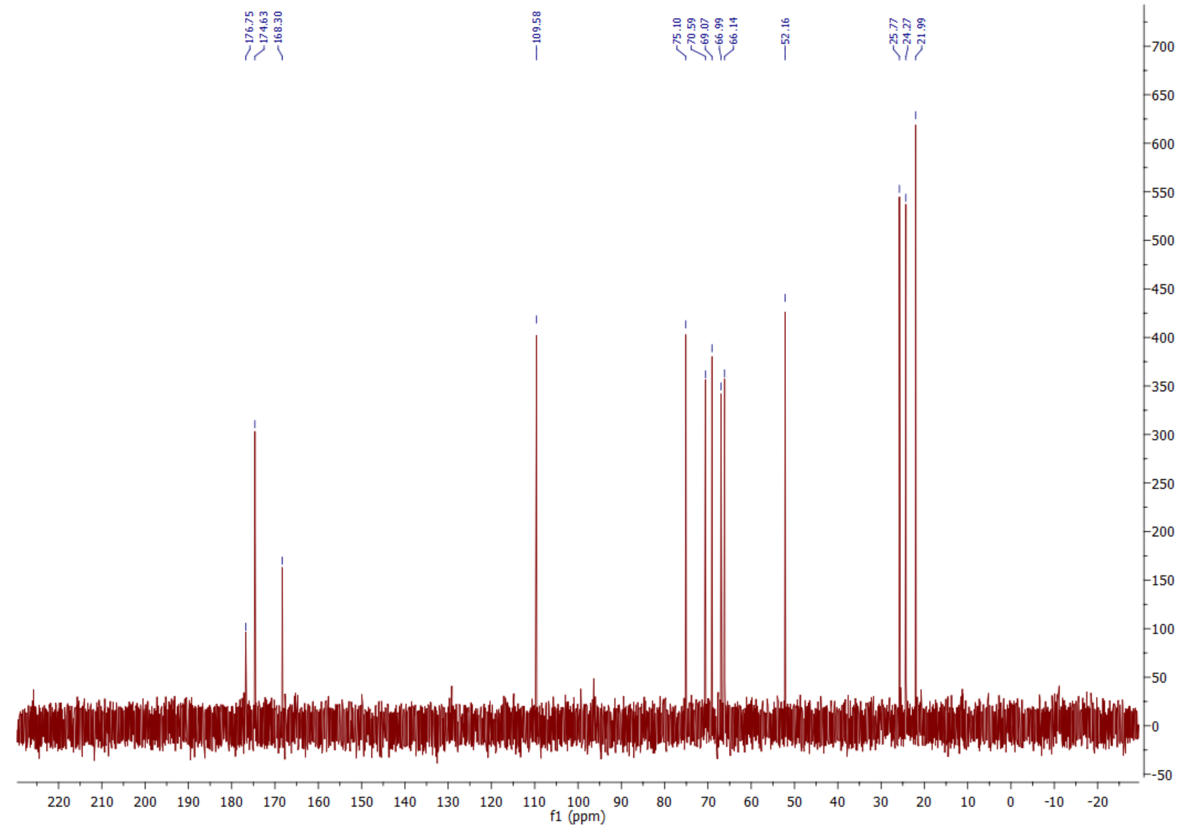

^{13}C NMR (100 MHz, D_2O): 5-Acetamido-3,5-dideoxy-8,9-*O*-isopropylidene-D-glycero-D-galactononulopyranosonate **(6)**

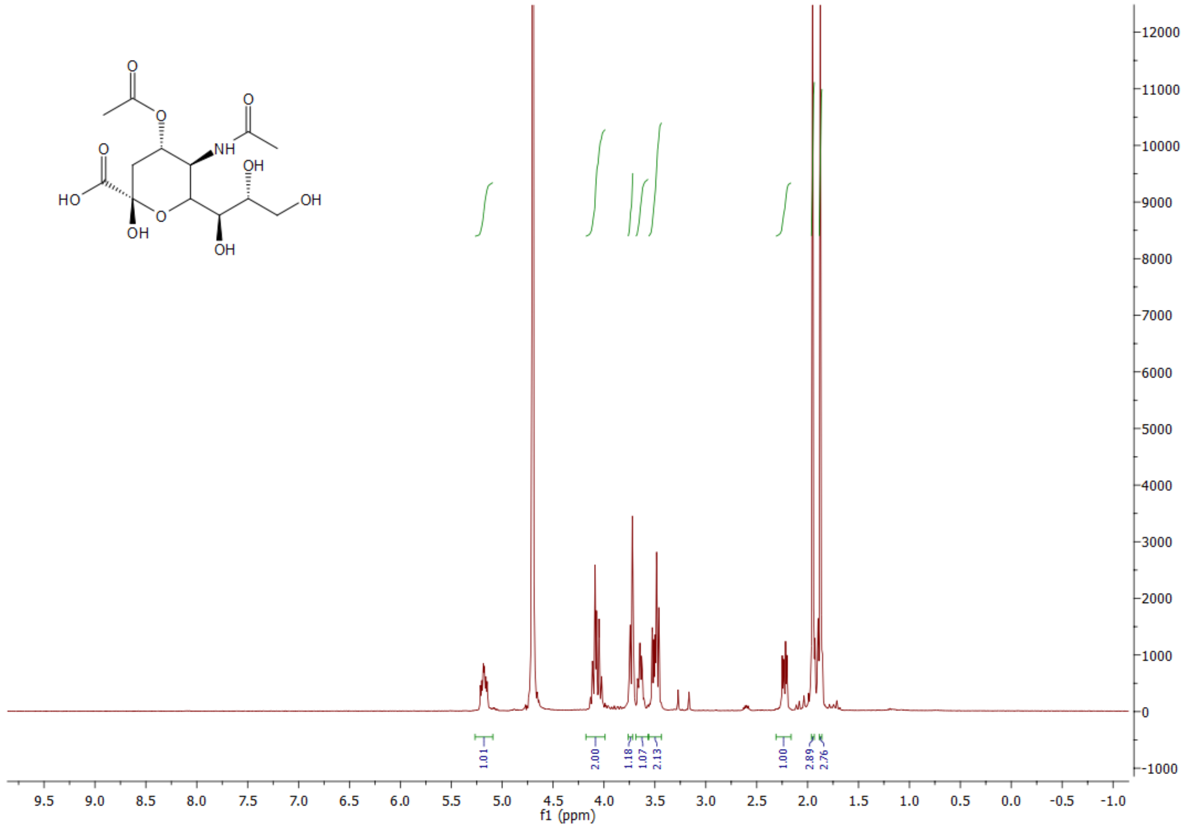

^{1}H NMR (400 MHz, D_2O): 5-Acetamido-3,5-dideoxy-4-*O*-acetyl-D-glycero-D-galactononulopyranosonate **(3)**

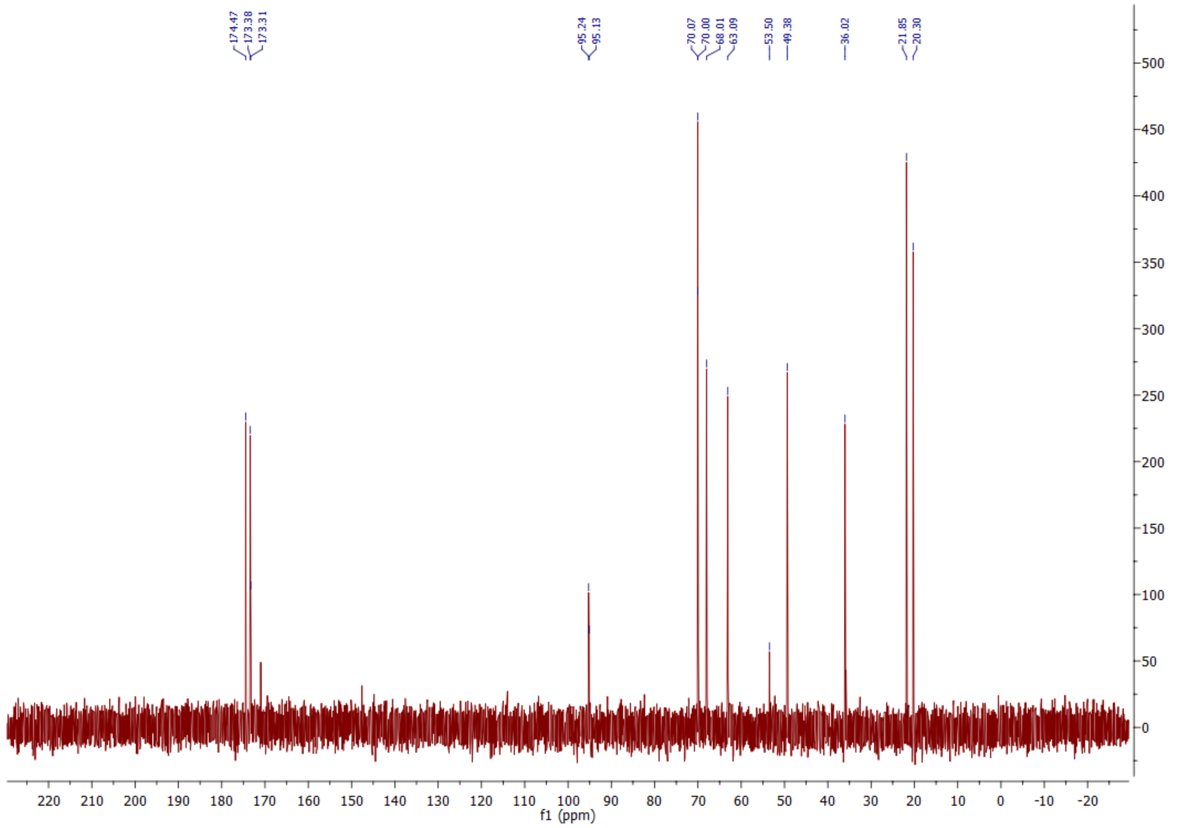

^{13}C NMR (100 MHz, D_2O): 5-Acetamido-3,5-dideoxy-4-*O*-acetyl-D-glycero-D-galactononulopyranosonate **(3)**

Quantitative NMR calibration curve

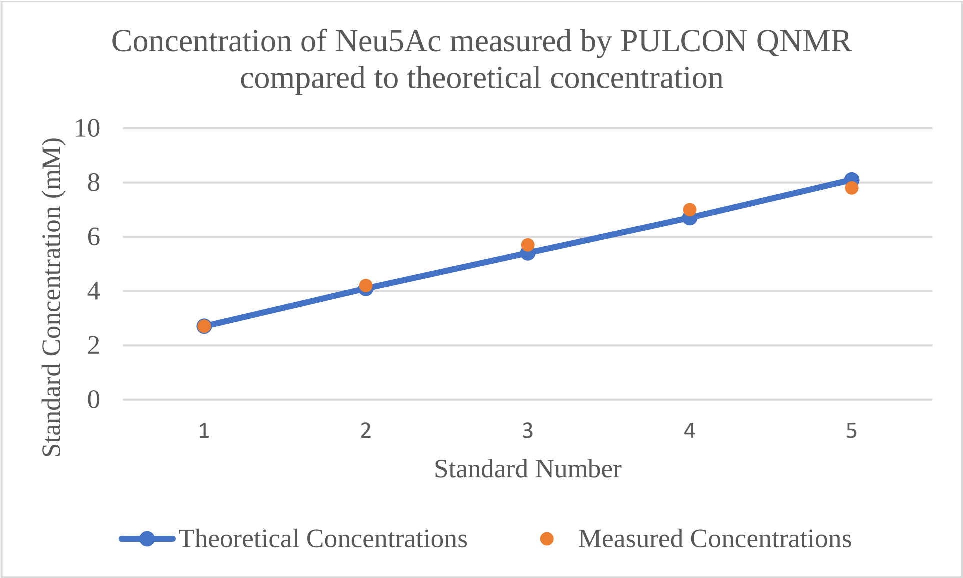

Concentration of Neu5Ac standards determined by QNMR (orange points) compared to the expected concentration of each standard (blue line)
